# Enhanced YOLO v3 for precise detection of apparent damage on bridges amidst complex backgrounds

**DOI:** 10.1038/s41598-024-58707-2

**Published:** 2024-04-15

**Authors:** Huifeng Su, David Bonfils Kamanda, Tao Han, Cheng Guo, Rongzhao Li, Zhilei Liu, Fengzhao Su, Liuhong Shang

**Affiliations:** 1 College of Transportation, Shandong University of Science and Technology, Qingdao, 266590 China; 2 Shandong Expressway Qingdao Development Co., Ltd., Qingdao, 266000 China

**Keywords:** YOLO v3 algorithm, Bridge disease detection, SENet, Spatial pyramid pooling, Engineering, Civil engineering

## Abstract

A bridge disease identification approach based on an enhanced YOLO v3 algorithm is suggested to increase the accuracy of apparent disease detection of concrete bridges under complex backgrounds. First, the YOLO v3 network structure is enhanced to better accommodate the dense distribution and large variation of disease scale characteristics, and the detection layer incorporates the squeeze and excitation (SE) networks attention mechanism module and spatial pyramid pooling module to strengthen the semantic feature extraction ability. Secondly, CIoU with better localization ability is selected as the loss function for training. Finally, the K-means algorithm is used for anchor frame clustering on the bridge surface disease defects dataset. 1363 datasets containing exposed reinforcement, spalling, and water erosion damage of bridges are produced, and network training is done after manual labelling and data improvement in order to test the efficacy of the algorithm described in this paper. According to the trial results, the YOLO v3 model has enhanced more than the original model in terms of precision rate, recall rate, Average Precision (AP), and other indicators. Its overall mean Average Precision (mAP) value has also grown by 5.5%. With the RTX2080Ti graphics card, the detection frame rate increases to 84 Frames Per Second, enabling more precise and real-time bridge illness detection.

## Introduction

As the number of highway bridges is increasing daily and in addition to many bridges being finished and placed into use, managing, and maintaining bridges has grown to be an increasingly difficult responsibility. In-service bridges will inevitably develop cracking, protective layer spalling, seepage and alkali, exposed tendons and corrosion, and other diseases due to the aging of concrete materials, severe vehicle overloading, poor operating environments, and other factors^1^. This is a great test of the bridge structure's durability and safety. The manual inspection process used in the traditional bridge inspection method has several drawbacks, including high subjectivity, a heavy workload, low efficiency, etc., and it eventually becomes unsatisfactory for the needs of the public. Bridge disease detection techniques based on digital image processing have garnered a lot of interest from the academic community with the advancement of sensor acquisition, information storage, and analysis technologies. A bridge crack detection approach based on binocular vision was proposed by Liu et al.^2^. The crack picture is subjected to Gaussian filtering, histogram equalization, edge detection, and binarization procedures, and the crack size is then determined by the binocular vision system. A method for classifying surface diseases of concrete bridges based on image eigenvalues was proposed by Chen et al.^3^; it involves extracting features from the disease images, such as texture, color moment, and gray scale histogram, and then using Support Vector Machine (SVM) to classify the disease images. SVM was utilized by Han et al.^4^ to categorize fractures according to attributes in the image connectivity domain.

These approaches, however, have limited application scenarios in bridge automated inspection since they mostly rely on human experience for sample feature extraction, and the generated features are still single-layer features without hierarchy. With the rapid advancement of intelligent inspection tools like drones^5^ and wall-climbing robots^6^ for assessing the structural appearance of bridges, deep learning based on convolutional neural networks (CNN)^7^, as a representative method is progressively becoming a research hotspot in academia and industry. This is because deep learning allows for the analysis of a large number of disease images gathered by intelligent inspection tools. In contrast to conventional machine learning algorithms, CNN can automatically extract the disease's structural properties, saving the manual labor that these techniques require. In contrast to conventional machine learning algorithms, CNN can automatically extract the disease's structural properties, saving the manual labor that these techniques require. Additionally, CNN is highly effective at eliminating background noise^8^, which may overcome noise-producing interferences such as stains, occlusion, uneven lighting, and other surface-level issues related to bridge construction. This ability is adequate to identify the intricacy of the back-end structure.

The CNN neural networks were created with the intention of identifying structural problems in bridges in 2021 through the extraction of crack and pothole features from the road surface, respectively. In order to identify road surface diseases, extract crack features, and extract pothole features, Sha et al.^8^ created three different types of CNN neural networks. They then demonstrated that the accuracy of CNN is adequate to meet the complex morphological characteristics of cracks, potholes, and other diseases. Han et al.^9^ used CNN to detect surface diseases on bridge structures, trained and adjusted the AlexNet model by migration learning, and created three different disease recognition models: corrosion, cracks, and flaws. Nevertheless, this type of CNN-based image classification technique has trouble defining the sliding window's size and handling disease images of varying sizes. Numerous target identification algorithms based on CNN are continuously receiving attention to increase the effectiveness of identifying and localizing different bridge diseases. The Faster R-CNN algorithm was utilized by Cha et al.^10^ to identify and classify five various types of defects, including corrosion on steel plates, cracks in concrete, and varying degrees of bolt damage. A Faster R-CNN approach was put forth by Xu et al.^11^ for the detection of reinforced concrete columns following an earthquake. An enhanced Faster R-CNN technique was presented by Xu et al.^11^ to identify various forms of damage in reinforced concrete columns following earthquakes. The single-stage target detection algorithms represented by SSD^12^ and Yolo^13^ are more appropriate for bridge automated inspection scenarios and avoid the step of generating candidate regions when compared to the two-stage target detection algorithm represented by Faster R-CNN^14^. They also achieve faster detection speeds. Using the YOLO v3 method, Zhang et al.^15^ achieved real-time identification of a variety of flaws on the bridge surface, including fractures, exposed tendons, spalling, etc. Target identification techniques for bridge defects in complex backgrounds still need to be developed immediately, as the current deep learning-based algorithms have not been updated to account for the characteristics of bridge surface defects.

The presented study enhances the Yolo v3 algorithm based on previous research to address issues with dense distribution and large-scale changes in bridge defects, as well as to increase the detection accuracy of bridge defects. Second, in order to train the model network, this paper creates a corresponding dataset for the task of bridge apparent disease detection in a complex background. It then categorizes the bridge detection images into three disease categories: exposed reinforcement, spalling, and water erosion. Finally, the test set confirmed the test set's practicality and accuracy of the model described in this study.

## YOLO v3 Algorithm

**Figure 1:**
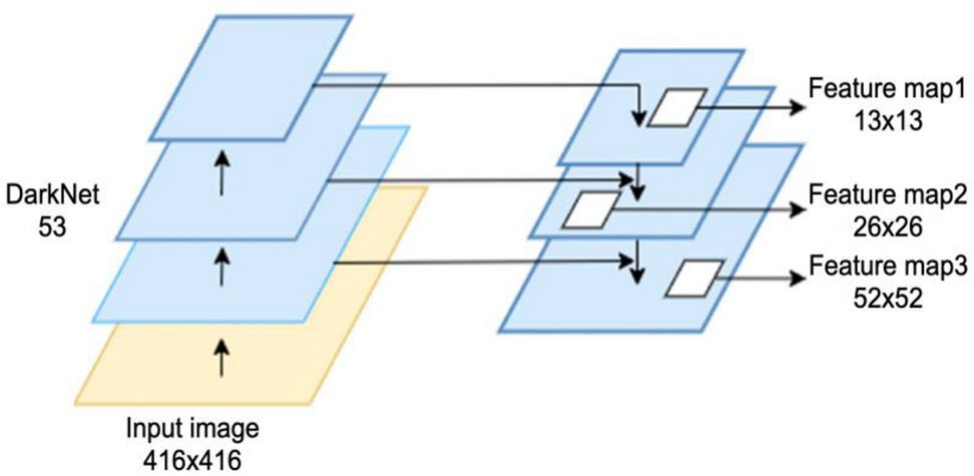
Feature pyramid network.

The YOLO v3 algorithm, which consists of the detection layer and the feature extraction backbone network, is a regression-based single-stage target detection algorithm that was proposed by Redmon et al.^13^. YOLO v3, which was influenced by ResNet's residual network^14^, creates the feature extraction backbone network darknet53 by adding residual units to the main network. This effectively addresses the gradient vanishing issue in deep neural networks and improves network model correctness. YOLO v3 employs down sampling rates of 1/32, 1/16, and 1/8 to yield, for the final three down samplings, 13 × 13, 26 × 26, and 1/8 of the down sampling rate, respectively. YOLO v3 produces 13 × 13, 26 × 13, and 26 × 16 down samples for the previous 3 down samplings, in that order. There are 3 distinct feature map scales: 26, 52 × 52. To combine the small-size feature maps, which offer the deep semantic information of the image, with the large-size feature maps, which offer a greater sensory field, the multi-scale prediction approach of Feature Pyramid Networks (FPN)^16^ is also utilized. The combination improves the ability to identify various disease sizes and enhances the details of disease characteristics across all scales, Fig. 1 shows this prediction.

**Figure 2:**
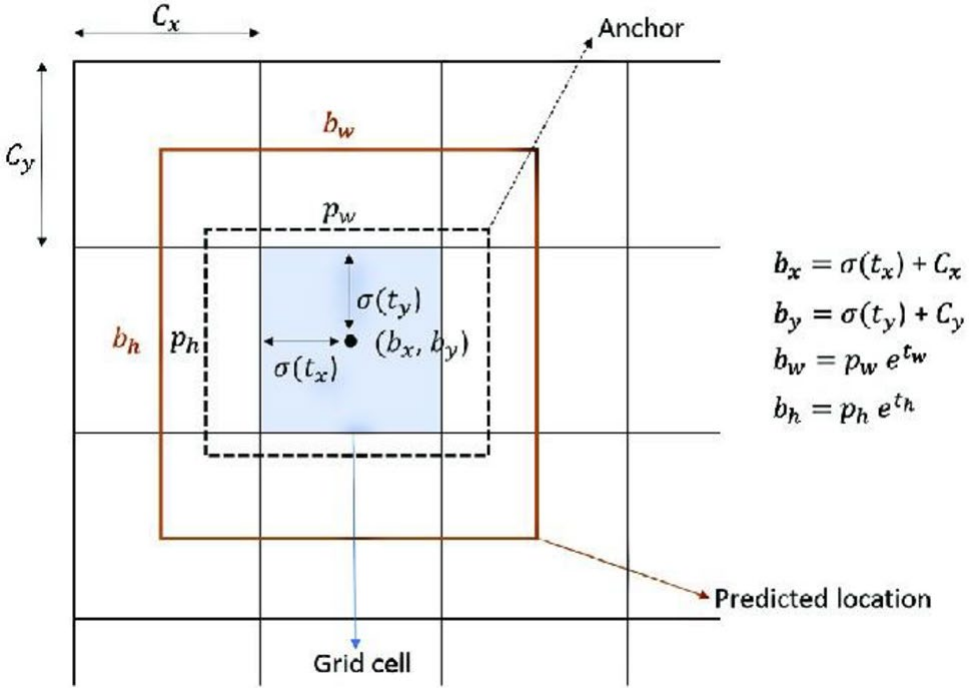
Bounding box with anchor and predicted position.

YOLO v3 separates the input illness image into S × S grids based on the feature map's size in the detection layer. Each cell is in charge of identifying the disease that falls into its center, and it produces multiple prediction boxes together with the confidence level of each prediction box. The parameters (*t*_*c*_*, t*_*x*_*, t*_*y*_*, t*_*w*_*, t*_*h*_) for each bounding box are as follows: (*t*_*x*_*, t*_*y*_) is the candidate box's center coordinate; (*t*_*w*_*, t*_*h*_) is its center point; and (c) is the confidence variable, as determined by the sigmoid function. The generated prediction coordinates are (*b*_*x*_*, b*_*y*_*, b*_*w*_*, b*_*h*_), where (*b*_*x*_*, b*_*y*_) are the center coordinates of the prediction bounding box and (*b*^*w*^*, b*^*h*^) are the width and height of the prediction box, as shown in Fig. 2. In the position prediction, using the coordinates of the upper left corner of a cell on each feature map as an example, each anchor box is predicted to be of size (*p*_*w*_*, p*_*h.*_). Following the output of several prediction frames, the low-confidence prediction frames will be eliminated, and non-great value suppression will be used to ultimately pinpoint the disease's site.

When directly used to the detection of bridge surface lesions in complex backgrounds, the YOLO v3 method still has the following shortcomings despite its excellent accuracy and speed. First, there are issues with the dense distribution of lesion scales on the bridge surface and the wide variation in lesion scales; second, YOLO v3 uses a multi-scale prediction method that fully utilizes the sensory field and semantic features; however, the extracted features' robustness is low, making it unsuitable for use in bridge lesion detection in complex backgrounds; and third, the intersection and merger ratio (IoU), which represents the detection effect between the prediction frame and the real labeling frame of the lesions, is still not very good when applied in a complex background. When the two frames do not intersect, the IoU cannot provide any adjustment gradient and the prediction accuracy of the disease's location will also decrease, even though it can reflect the detection effect between the prediction frame and the real labeling frame of the disease. Therefore, the research conducted in this paper enhances the YOLO v3 algorithm by merging the characteristics of bridge diseases.

## Enhanced YOLO v3 algorithm

The algorithm's specific improvements can be broken down into four main components: the spatial pyramid pooling module, the embedded feature extraction network in SENet, the use of a better-localized loss function, and the use of anchors that cluster their own dataset, thereby increasing the algorithm's overall detection accuracy.

### Feature extraction network embedded in SENet

**Figure 3:**
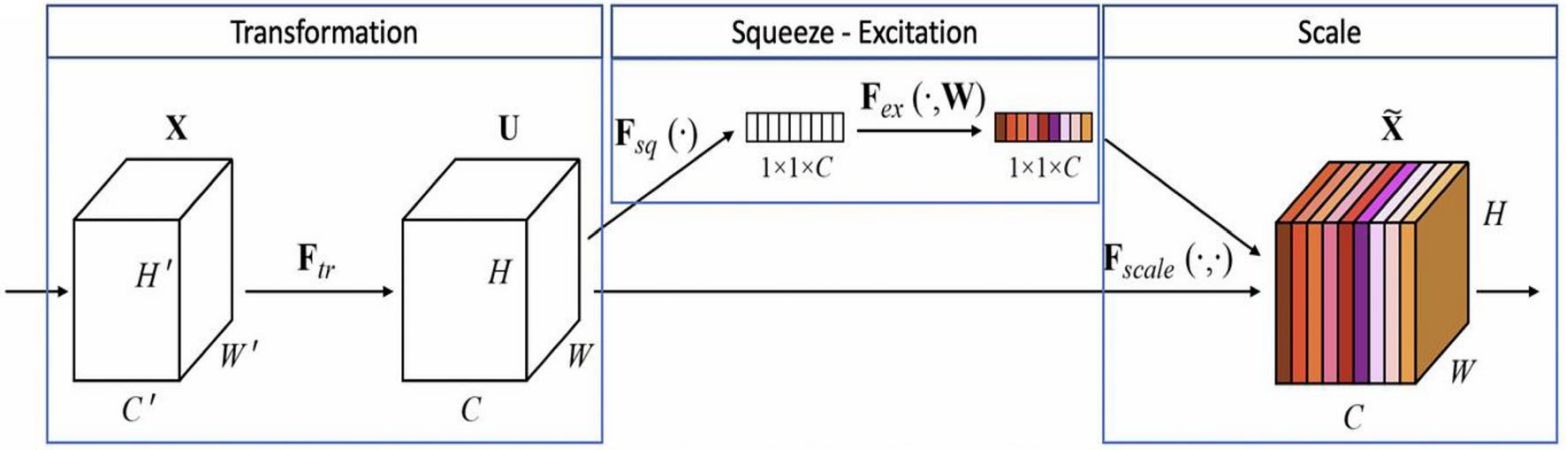
Squeeze and excitation network structure.

To address the issues of disease, overlap and dense distribution in bridge disease detection, this paper incorporates the SE attention mechanism structure in front of the three detection layers of YOLO v3, respectively, so that the network produces the channel weights and re-calibrates the channels, and outputs the special features with stronger expression ability. The network structure of SENet, an attention mechanism structure suggested by Lin et al.^16^, is displayed in Fig. 3^17^. Squeeze and excitation are the two primary activities that make up the SE module. First, a feature map X ∈ *R*^*H′*× *W′* × *C′*^ is input, and after being converted by F_tr_, it yields a feature map *U* ∈ *R*^*H*×*W*×*C*^, where *U* = [*u*_*1*_*,u*_*2,*_*…,u*_*c*_]. The feature map *U* is next subjected to global average pooling, which produces a feature map *Z*_c_ with a dimension of 1 × 1 × C, where C is the number of channels.

1 $${Z}_{c}={F}_{sq}\left({u}_{c}\right)=\frac{1}{H \times W} {\sum }_{i=1}^{H}{\sum }_{j=1}^{W}{u}_{c} \left(i,j\right)$$

The generated feature maps are passed through two fully connected layers, dimensionally reduced, and then upgraded, and the compression rate r is set to 16. Next, the sigmoid activation function is used to obtain the corresponding weights S = [s1, s2,…, sc] between each channel in order to further extract the inter-channel correlation.

2 $$S={F}_{ex}\left({Z}_{c},W\right)=sigmoid\left({W}_{2}ReLU\left({W}_{1}{Z}_{c}\right)\right)$$

3 $${W}_{i}\in {R}^{\frac{c}{r}\times c},{W}_{2}\in {R}^{c\times \frac{c}{r}}$$

Finally, the weights are updated by multiplying each channel with the corresponding weights to obtain the updated output $\widetilde{X}=\left[{\widetilde{x}}_{1},{\widetilde{x}}_{2}, \dots , {\widetilde{x}}_{c}\right]$.

4 $${\widetilde{x}}_{c}={F}_{scale}\left({u}_{c }{s}_{c}\right)={s}_{c}\cdot {u}_{c}$$

During the process of generating feature maps, the SE attention mechanism directs the network's attention towards various types of bridge disease features. Simultaneously, it improves the disease features' semantic information through the form of network self-attention, suppresses the complex background information of the concrete bridge deck, and solves the problem of the bridge's poor recognition accuracy when the apparent disease is densely distributed.

### Space pyramid pooling module

**Figure 4:**
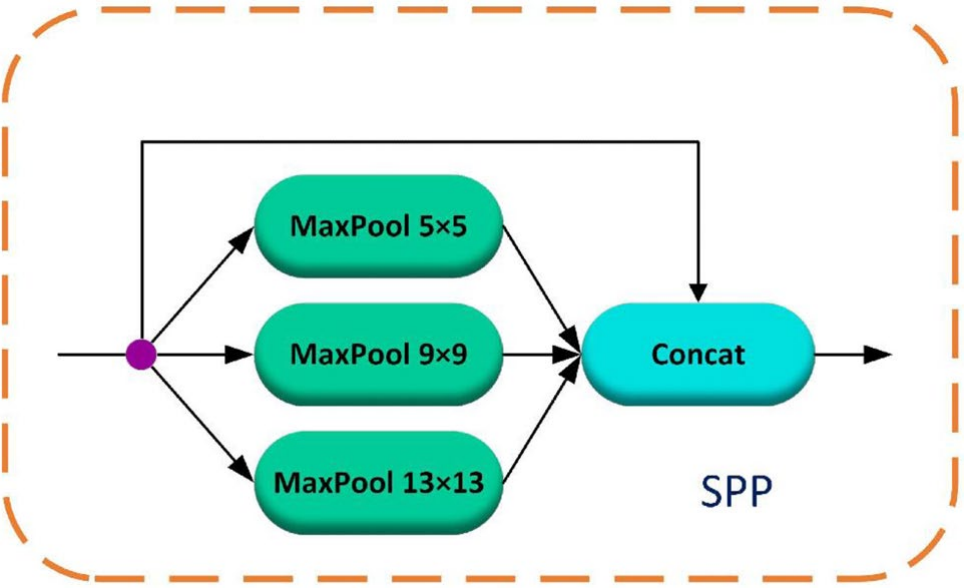
Spatial pyramid pooling structure.

To deal with the problem of bridge diseases exhibiting significant variations in the photos obtained by various bridge inspectors, it is difficult to effectively extract the disease features better. To further improve the feature map data, we're introducing the Spatial Pyramid Pooling (SPP) module in this paper. He et al.^17^ introduced the SPP approach as a solution to the neural network problem involving different picture size inputs. The feature maps output from the backbone network darknet53 is passed through three maximum pooling layers with convolutional kernel sizes of 5 $\times$ 5, 9 $\times$ 9, and 13 $\times$ 13, respectively. To further enrich the structural feature representations by fusing the local feature information of the disease with the global feature information, which is especially useful for the detection of different sizes of diseases and improves the overall recognition accuracy of the disease. The structure of the SPP module is shown in Fig. 4.

### Localization loss function

IoU, serving as one of the most widely utilized performance metrics in target detection is based on the Jaccard index to measure the overlapping area that lies between the ground truth and the predicted bounding boxes^18^. It represents the intersection and concurrency ratio of the true labeled disease frames to the predicted disease frames, which is calculated as shown in Eq. (5):

5 $${\text{IoU}}=\frac{\left|{B}_{pred}\cap {B}_{true}\right|}{\left|{B}_{pred} \cup {B}_{true}\right|}$$

where: ${B}_{pred}$ denotes the bridge disease prediction box; ${B}_{true}$ denotes the bridge disease real labeling frame, the size of IoU reflects the detection effect of the disease. However, when the disease prediction frame and the real frame are not intersected, the IoU is 0, which cannot reflect the size of the distance between the two at this time, resulting in the inability to propagate the adjustment gradient. To address this issue, this paper introduces the newly proposed CIoU^19^ localization function, compared with IoU, CIoU considers the distance of the centroid between the disease prediction frame and the real frame, the overlap ratio, and the aspect ratio, which makes the bounding box regression more stable when the gradient is decreasing. The calculation of CIoU is shown in Eq. (6):

6 $${\text{CIoU}}={\text{IoU}}-\frac{{\rho }^{2}\left(b,{b}^{gt}\right)}{{c}^{2}}-\alpha \beta$$

where: b and ${b}^{gt}$ represent the centroids of the disease prediction frame and the real frame, respectively; ${\rho }^{2}\left(b,{b}^{gt}\right)$ represents the Euclidean distance between the disease prediction frame and the center frame; c denotes the diagonal distance of the smallest area that can contain both the disease prediction frame and the real frame; α is a trade-off parameter; and β is used as a measure of the consistency of the aspect ratio.

### Clustering anchor frames by k-means algorithm

K-means clustering, which falls in the category of “unsupervised learning algorithms,” apportions a dataset into clusters^20^. While YOLO v3 is the object detection method, it applies the k-means algorithm in the estimation of heights and widths of the bounding boxes that have been predicted^21^.

YOLO v3 predicts the localization of bounding boxes by using anchor boxes. However, on the self-constructed bridge dataset, the scale varies greatly among the lesions and the aspect ratios are significantly different, so it is necessary to cluster its own anchor boxes, and a total of nine groups of a priori boxes are generated after K-means clustering, which include (11 × 34), (33 × 15), (16 × 72), (41 × 52), (88 × 25), (25 × 163), (67 × 175), (120 × 70), (190 × 161).

**Figure 5:**
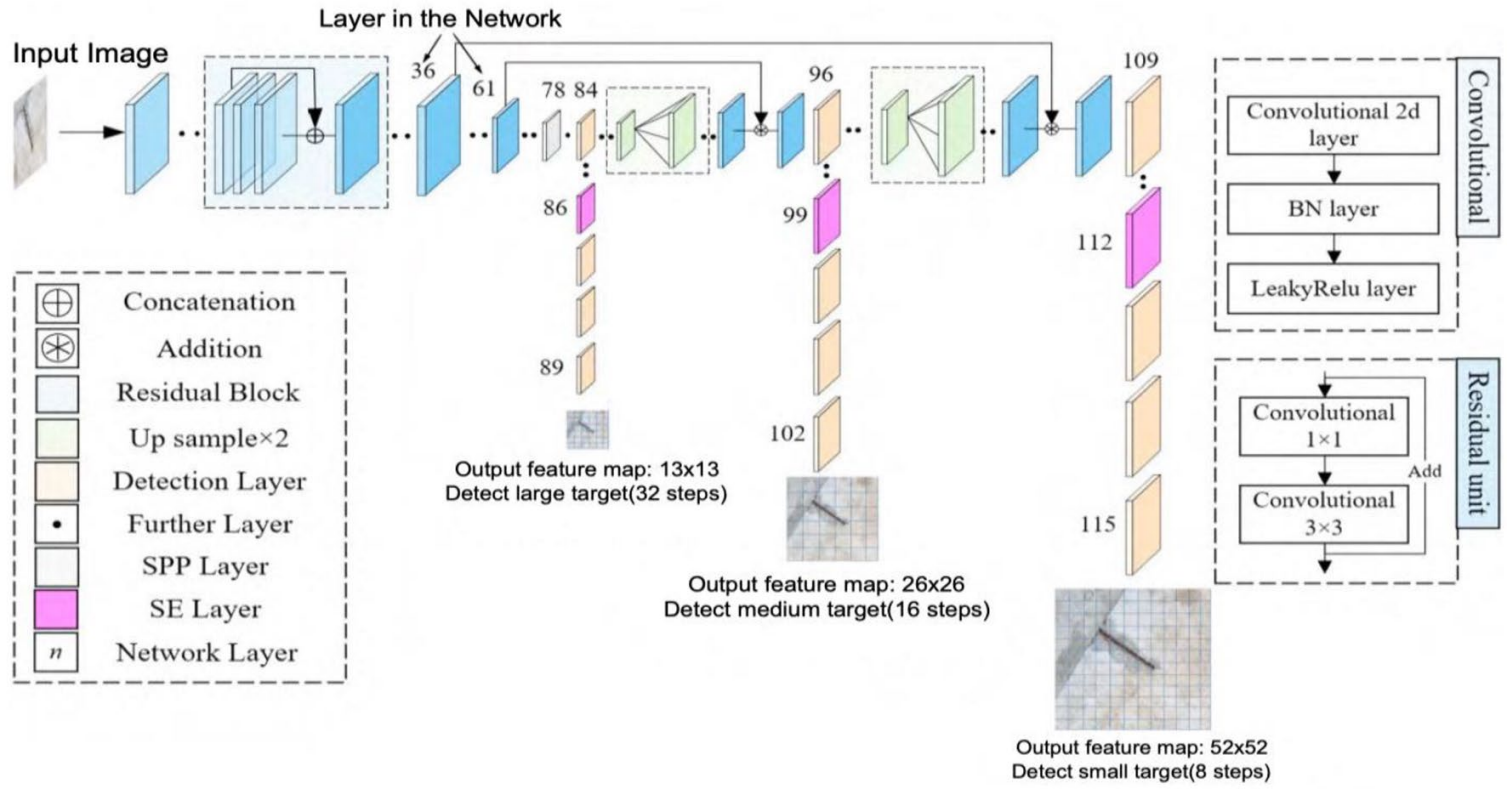
Enhanced YOLO v3 architecture.

After incorporating the aforementioned techniques, the structure of the enhanced YOLO v3 algorithm is displayed in Fig. 5. The SPP module is situated in the 78th to 83rd layers of the network, while the SE attention layer is embedded in three detection layers, which are located in the 86th, 99th, and 112th layers of the darknet53 network.

## Bridge disease dataset and hyperparameter setting

### Bridge disease image dataset

**Figure 6:**
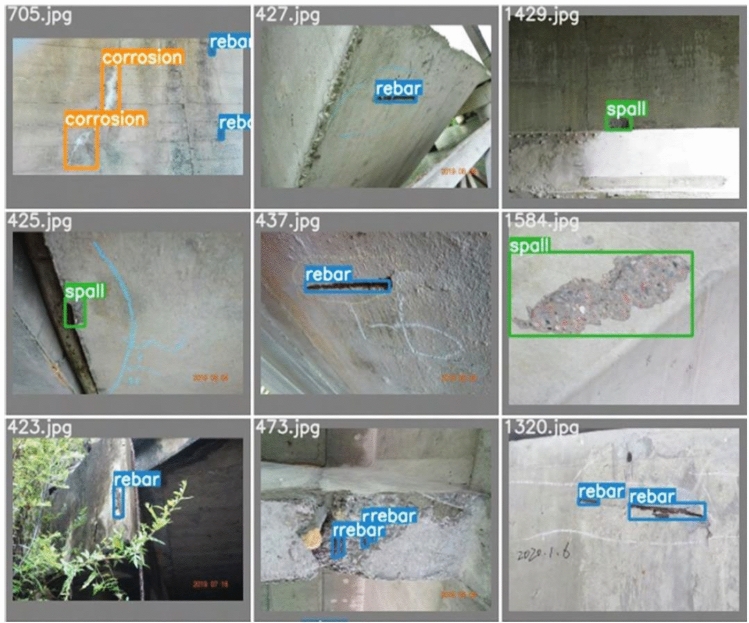
Bridge apparent damage ground truth.

2603 real values of the target lesions were tagged after a total of 1363 bridge inspection pictures were screened for the three most prevalent types of concrete spall, rebar, and corrosion in the bridge inspection reports. The idea behind screening photographs is that the disease areas should be clearly visible and have a high quality. These images of bridge diseases were captured by several bridge inspectors. Each image's disease areas were tagged using the open-source labelImg tool, a popular tool for annotating images^22^; Fig. 6 displays an example of partially labeled ground-truth values.

### Introduction to the experimental environment

The experiments were conducted using the open source Pytorch 1.6.0 deep learning framework with Intel® Core ™ i9-9900KF CPU, Ge-Force RTX 2080Ti graphics card, 64 GB RAM, ubuntu 18.04 LTS, Python 3.7.7, CUDA 10.2, cuDNN 7.6.5, OpenCV 4.4.0 environment.

### Experimental parameter setting

The learning rate ${\eta }_{t}$ can be expressed as shown in Eq. (7), where t is the batch size and T is the number of whole epoch rounds. The network training uses a stochastic gradient descent algorithm with momentum, and the momentum factor is 0.9. The initial learning rate is set to 0.01, the final learning rate to 0.000 5, and the learning rate decay strategy is the cosine annealing strategy^23^.

7 $${\eta }_{t}=\frac{1}{2}\left(1+{\text{cos}}\left(\frac{t\pi }{T}\right)\right)\eta$$

There will be 300 training rounds with a batch size of 16. The model's input image size is 416 × 416, with 80% of the photos functioning as the training set and 20% functioning as the test set. In order to improve the model's capacity for generalization, data enhancement is applied to the training set throughout the training process. This results in a total of 5455 training sets after enhancement. Data enhancement techniques include random cropping, panning, horizontal flipping, vertical flipping, etc. This paper employs mosaic^24^ data enhancement to further improve the small target recognition effect. The process of splicing together four randomly cropped photos enhance the background of the object to be detected and boosts the effectiveness of small target detection.

## Analysis of experimental results

### Performance evaluation indicators

In this paper, the evaluation indexes commonly used in target detection are selected for analysis, and the statistical indexes used are precision, recall, AP of each type of disease, mean (mAP) and Frames Per Second (FPS) were evaluated. Precision entails the quantity of samples that are labelled as positive and are truly positive while recall is the quantity of positives that have been correctly classified. Average precision, which is the area below the precision-recall curve measures the algorithm's accuracy in the identification of relevant points that it indicates as positive while mAP averages the APs in all classes^25^. The detection results can be categorized into four types: true positive case (TP), true negative case (TN), false positive case (FP), and false negative case (FN), and the precision rate and the detection rate are defined in Eqs. (8–9):

8 $${\text{Precision}}=\frac{{\text{TP}}}{{\text{TP}}+{\text{FP}}}$$

9 $${\text{Recall}}= \frac{{\text{TP}}}{{\text{TP}}+{\text{FN}}}$$

The AP and mAP are defined as in Eqs. (10)–(11):

10 $${\text{AP}}={\int }_{0}^{1}{\text{P}}\left(\mathrm{ R }\right){\text{dR}}$$

11 $$mAP=\frac{\sum_{{\text{i}}=1}^{{\text{N}}}{{\text{AP}}}_{{\text{i}}}}{{\text{N}}}$$

where R (in Eq. 10) is the recall and N (in Eq. 11) is the number of categories for diseases. The number of images that a Graphic Processing Unit (GPU) can identify in a second is known as FPS. mAP@0.5 metrics and detection speed fps are the primary metrics used in this paper to evaluate the model.

### Analysis and comparison of experimental results

#### Comparison of experimental results between this paper's algorithm and the YOLO v3 algorithm

Table 1 compares the performance of the original YOLO v3 algorithm with the enhanced YOLO v3 method. It demonstrates that great detection accuracy for concrete spalling and exposed reinforcement damage is achieved, but relatively low detection accuracy for water penetration damage. This is because the water erosion damage is unevenly distributed at the bottom of the beams and is relatively less different from the background, meaning that the classification accuracy of the overall detection is relatively low. In contrast, the spalling and exposed reinforcement are relatively more different from the background and the robustness of the extracted features is better. With a 5.5% increase in the mAP value (mAP% increase from 0.743 to 0.789), the enhanced YOLOv3 algorithm shown in this paper performs better in its accuracy at bridge apparent disease detection.

**Table 1:** Comparison of YOLO v3 and enhanced YOLO v3.

Methods	Spall	Corrosion	Rebar	mAP%	FPS
YOLO V3	0.794	0.634	0.800	0.743	86
Our methods	0.860	0.684	0.850	0.789	84

**Figure 7:**
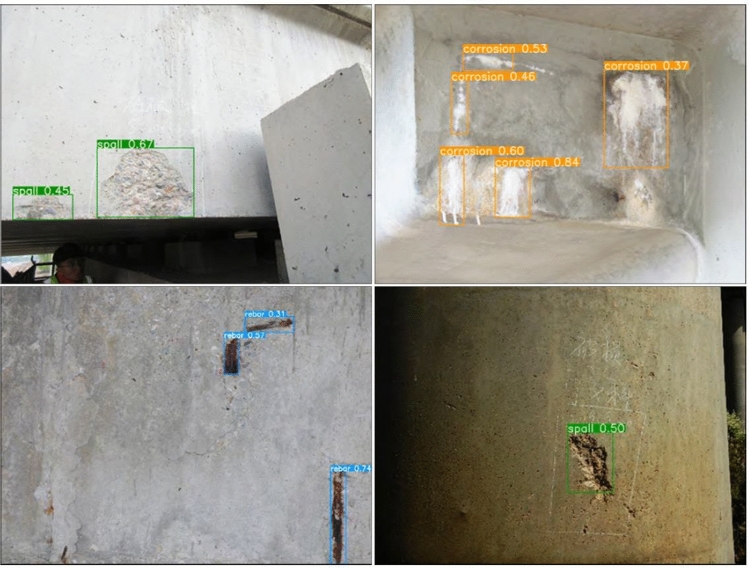
Detect disease results by using YOLO v3 algorithm.

**Figure 8:**
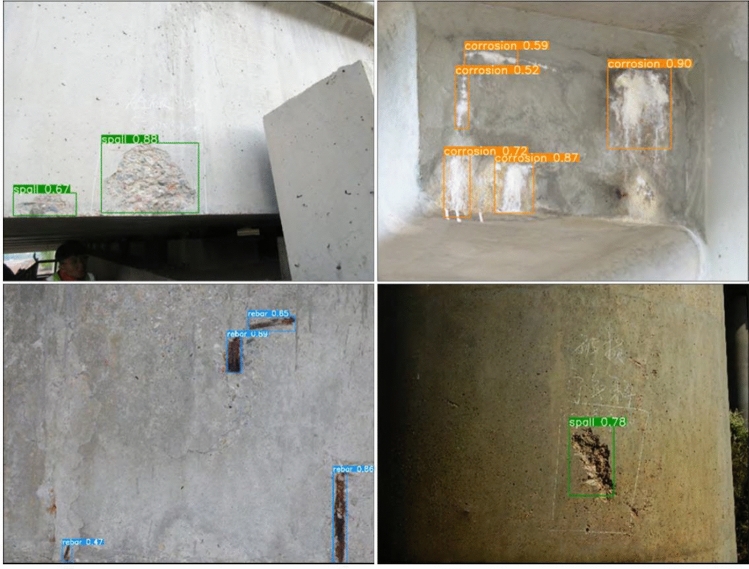
Detect disease results by using enhanced YOLO v3 algorithm.

In the next section, this paper will show the actual detection effect of three different types of concrete spalling, water erosion, and exposed reinforcement in the complicated background. Figures 7 and 8 display the detection effect of YOLO v3 and the enhanced YOLO v3.

Figures 7 and 8 demonstrate that the enhanced YOLO v3 algorithm has improved disease identification precision. It can now accurately identify water erosion and concrete spalling diseases, as well as exposed reinforcement diseases of bridges with relatively small target sizes. Additionally, it still exhibits better disease identification precision in low light, which can effectively reduce the rate of misdetection and omission of diseases caused by background complexity, dense distribution, light conditions, and small disease sizes. Because of the small lesions, dense distribution, complicated background, and lighting, it can significantly improve the misdetection and omission scenario.

#### Analysis of ablation experiment results

For ablation experiments, the enhanced YOLOv3 algorithm provided in this paper is divided into 5 groups of distinct network models in order to further investigate the impact of each added network structure branch on the model as a whole. The experiments are divided into five groups: group 1 is the YOLO v3 algorithm; group 2 improves the mosaic data on the training data; group 3 adopts the CIoU localization loss function based on group 2; group 4 adds the SPP spatial pyramid pooling module; and the last group embeds the SE attentional mechanism on the basis of group 4 i.e., group 5 is the enhanced YOLO v3 algorithm proposed in this paper. Table 2 displays the particular experimental results for the YOLO v3 algorithm.

**Table 2:** Comparison of experimental results of ablation.

Model	Mosaic	CIoU	SPPNET	SENet	Spall	Corrosion	Rebar	mAP%	FPS
V3(1)	×	×	×	×	0.794	0.634	0.800	0.743	86
V3(2)	√	×	×	×	0.825	0.616	0.824	0.755	85
V3(3)	√	√	×	×	0.858	0.639	0.828	0.775	87
V3(4)	√	√	√	×	0.824	0.672	0.859	0.785	85
V3(5)	√	√	√	√	0.859	0.684	0.850	0.798	84

Table 2 displays the results of the ablation experiments. The original version of YOLO v3 in Group 1 achieved a mAP value of 74.3% with a detection speed of 86 fps; Group 2 enhanced the model's ability to generalize by using mosaic data enhancement, which led to an overall 1.2% improvement in its mAP value; for the model in Group 3, the adoption of the CIoU localization loss function, which better describes the distance between the prediction frame and the real disease labeling frame, further accelerated the model's convergence speed, improving both detection accuracy and detection speed; while the fourth group of experiments embeds a spatial pyramid pooling module, which further solves the problem of large scale changes of diseases in different detection images, especially for water erosion diseases, and improves its AP value by 3.3%, and at the same time the detection speed slightly decreases with the introduction of the network module; the last group of models has an overall improvement of mAP value by 1.2% due to the CIoU localization loss function. The last group, i.e., the enhanced YOLO v3 algorithm proposed in this paper, further enhances the semantic information of the disease features by embedding the SENet attention mechanism, which achieves a mAP value of 79.8%, and at the same time increases the number of model parameters, so the final detection speed is 84 fps.

In summary, every suggested improvement strategy has a certain outcome. The AP values of the three distinct bridge structural diseases, namely spalling, water erosion, and exposed reinforcement—are increased by 6.5%, 5.0%, and 5.0%, respectively, in comparison to the original YOLO v3 algorithm. This represents a significant overall improvement. In terms of detection speed, the addition of the SPPNet and SENet modules, which also provide more model parameters, results in a tiny decrease in detection speed fps but leaves it at 84fps, allowing for the more accurate and real-time detection of bridge damage.

#### Comparison of the results of this paper's algorithm with other target detection algorithms

This paper employs the Faster R-CNN detection algorithm and the SSD detection method to perform comparison experiments in order to assess the enhanced YOLOv3 algorithm more thoroughly. Faster R-CNN is a cutting-edge algorithm that has shown good performance in object-detection tasks while SSD is an algorithm with multi-scaled features and anchor boxes that detect multi-sized objects in a scene in one shot^26^. The experimental findings are displayed in Table 3. As Table 3 shows, the two-stage Faster R-CNN method achieves 70.9% of the mAP value; however, its detection speed is limited to 15 fps because it must generate the target candidate region. In contrast, the SSD and YOLO algorithms achieve faster detection speeds by predicting the object directly because the intermediate step of generating a candidate region is eliminated; in this case, the YOLO v3 algorithm performs better in terms of speed and accuracy. YOLO v3 algorithm operates more quickly and accurately as is the case of the lighter version, YOLO v5 which has high accuracy in detecting structural defects^27^. The paper presents an enhanced YOLO v3 algorithm that increases the average detection accuracy by 5.5%. This makes it more appropriate for use in complex backgrounds where apparent bridge damage needs to be detected. Additionally, the detection speed fps of the enhanced algorithm is only 2 fps slower than the original YOLO v3, meaning that it can still identify bridge damage with greater accuracy and at a high speed, a speed that can enable it to detect defects in real time^28^.

**Table 3:** Comparison of enhanced YOLO v3 with other target detection algorithms.

Model	Spall	Corrosion	Rebar	mAP%	FPS
YOLOV3	0.794	0.634	0.800	0.743	86
Faster R-CNN	0.770	0.581	0.766	0.709	15
SSD-512	0.822	0.597	0.648	0.689	55
Our methods	0.859	0.684	0.850	0.798	84

## Conclusion

An enhanced YOLO v3-based bridge apparent disease recognition method is proposed, which, by introducing the SE attention mechanism and the SPP module to generate more informative feature maps, effectively suppresses the background information on the surface of concrete bridges in complex scenarios. In addition, the network is trained with a better localization loss function and anchor frames, which effectively improves the bridge lesion leakage detection caused by the background complexity, dense distribution, lighting conditions, and small lesion size.

The mAP value of the enhanced YOLO v3 algorithm is 79.8%. Its mAP value is 5.5% higher than that of the previous YOLO v3 algorithm, and it maintains a detection speed of 84fps. This means that it can identify bridge diseases in complex backgrounds more rapidly and reliably.

## Data Availability

The datasets used and/or analyzed during the current study are available under dataset name “CODEBRIM”: https://doi.org/10.5281/zenodo.2620293.
